# A cluster-randomized trial to reduce major perinatal morbidity among women with one prior cesarean delivery in Québec (PRISMA trial): study protocol for a randomized controlled trial

**DOI:** 10.1186/s13063-017-2150-x

**Published:** 2017-09-20

**Authors:** N. Chaillet, E. Bujold, B. Masse, W. A. Grobman, P. Rozenberg, J. C. Pasquier, A. Shorten, M. Johri, F. Beaudoin, H. Abenhaim, S. Demers, W. Fraser, M. Dugas, S. Blouin, E. Dubé, R. Gauthier, PRISMA Trial Research Group, Nils Chaillet, Emmanuel Bujold, Suzanne Demers, Marylène Dugas, Bruno Piedboeuf, Mira Johri, François Beaudoin, Robert Gauthier, Benoit Masse, François Audibert, Louise Duperron, Marie Josée Bédard, François Champagne, Diane Francoeur, William D. Fraser, Jean-Charles Pasquier, Haim Abenhaim, Robert Gagnon, Patricia Monnier, Isabelle Girard, Guy-Paul Gagné, William A. Grobman, Allison Shorten, Patrick Rozenberg, Jennifer Blake, Isabelle Girard, Diane Francoeur, Andrée Gagnon, Luisa Ciofani, Marie-Eve St Laurent, Daniel Riverin, Benoit Masse, Eric Dubé, Simon Blouin, Robert Platt, Shiliang Liu, Fernando Althabe

**Affiliations:** 1 Department of Obstetrics and Gynaecology, Laval University, Quebec, QC Canada; 2 Department of Epidemiology and Biostatistics, University of Montréal, Montréal, QC Canada; 3 Department of Obstetrics and Gynaecology, Northwestern University, Chicago, IL USA; 4 Service de gynécologie obstétrique et médecine de la reproduction, Centre hospitalier intercommunal de Poissy/Saint-Germain-en-Laye, 10, rue du Champ-Gaillard, 78303 Poissy, France; 5 Department of Obstetrics and Gynecology, Sherbrooke University, Quebec, QC Canada; 6 UAB School of Nursing, University of Alabama, Birmingham, AL USA; 7 University of Montreal, Hospital Research Center (CRCHUM), Montreal, QC Canada; 8 Department of Obstetrics and Gynecology, University of Montreal, Montreal, QC Canada; 9 Department of Obstetrics and Gynecology, McGill University, Jewish Hospital, Montreal, QC Canada; 10 Population Health and Optimal Health Practices Research Unit, CHU de Québec Research Centre, Quebec, QC Canada; 11 Faculté de Médecine, Département d’Obstétrique & Gynécologie, Université Laval, Centre de recherche du CHUQ, 2705, Boul. Laurier, local T-R-92, Quebec, QC G1V 4G2 Canada

**Keywords:** VBAC, Uterine rupture, Intrapartum management, RCT

## Abstract

**Background:**

Rates of cesarean delivery are continuously increasing in industrialized countries, with repeated cesarean accounting for about a third of all cesareans. Women who have undergone a first cesarean are facing a difficult choice for their next pregnancy, i.e.: (1) to plan for a second cesarean delivery, associated with higher risk of maternal complications than vaginal delivery; or (b) to have a trial of labor (TOL) with the aim to achieve a vaginal birth after cesarean (VBAC) and to accept a significant, but rare, risk of uterine rupture and its related maternal and neonatal complications. The objective of this trial is to assess whether a multifaceted intervention would reduce the rate of major perinatal morbidity among women with one prior cesarean.

**Methods/design:**

The study is a stratified, non-blinded, cluster-randomized, parallel-group trial of a multifaceted intervention. Hospitals in Quebec are the units of randomization and women are the units of analysis. As depicted in Figure 1, the study includes a 1-year pre-intervention period (baseline), a 5-month implementation period, and a 2-year intervention period. At the end of the baseline period, 20 hospitals will be allocated to the intervention group and 20 to the control group, using a randomization stratified by level of care. Medical records will be used to collect data before and during the intervention period. Primary outcome is the rate of a composite of major perinatal morbidities measured during the intervention period. Secondary outcomes include major and minor maternal morbidity; minor perinatal morbidity; and TOL and VBAC rate. The effect of the intervention will be assessed using the multivariable generalized-estimating-equations extension of logistic regression. The evaluation will include subgroup analyses for preterm and term birth, and a cost-effectiveness analysis.

**Discussion:**

The intervention is designed to facilitate: (1) women’s decision-making process, using a decision analysis tool (DAT), (2) an estimate of uterine rupture risk during TOL using ultrasound evaluation of low-uterine segment thickness, (3) an estimate of chance of TOL success, using a validated prediction tool, and (4) the implementation of best practices for intrapartum management.

**Trial registration:**

Current Controlled Trials, ID: ISRCTN15346559. Registered on 20 August 2015.

**Electronic supplementary material:**

The online version of this article (doi:10.1186/s13063-017-2150-x) contains supplementary material, which is available to authorized users.

## Background

Compared to historical rates, the rate of cesarean deliveries is high in industrialized countries. In Canada, these rates increased from 21.2 to 28.0% between 2000 and 2008 and remained stable since 2011 [1–3]. Previous cesarean deliveries alone are an indication for over 30% of all cesareans in Canada. Every year, over 30,000 women in Canada who have undergone a cesarean delivery will be faced with a difficult choice for their next pregnancy, i.e.: (1) plan for a second cesarean delivery, associated with a higher risk of maternal complications than a vaginal delivery [4, 5], or (2) have a trial of labor (TOL) to achieve a vaginal birth after cesarean (VBAC) and accept a significant, but rare, risk of uterine rupture [6–9], which constitutes a principal complication related to a TOL [10–16]. However, although an elective repeat cesarean (ERC) may prevent uterine rupture in most cases, this intervention is associated with a higher risk of maternal and perinatal complications, particularly compared to a VBAC [4, 16–21]. The choice of the mode of delivery among women with a prior cesarean delivery constitutes an important public health issue within the current context, in which health professionals hesitate, due to a variety of reasons – including the risk of litigation – to recommend a TOL in the absence of validated and effective methods that can predict the risk of uterine rupture [22, 23].

Recently, new clinical tools have been developed to assist in women’s decision-making [24], including those that help estimate the chance of TOL success [25–27], and the risk of uterine rupture [16, 28–30]. Several RCTs performed in pregnant women and a recent meta-analysis [24], demonstrated that decision analysis tools (DAT) are effective in improving women’s knowledge; reducing anxiety and decisional conflict; and guiding women’s final choice during the decision-making process [31–44]. Based on a study involving 9616 women attempting VBAC in United States, Grobman et al. developed a nomogram to assess the chances of TOL success based on *antepartum* clinical factors and factors available upon admission at the time of delivery [25, 26]. Two observational studies and the recent statements of the NIH Consensus Development Conference confirmed the validity of this instrument in North America [16, 45–47]. Grobman et al. also highlighted that a prediction model for TOL success provides also additional information regarding the chance of TOL-related morbidity. They showed that maternal and neonatal morbidity is not greater for those women who undergo TOL than for those who undergo an elective repeat cesarean delivery when the chances of VBAC success are at least 70% [48]. Finally, two observational studies in Québec, along with a meta-analysis, assessed the effectiveness of measuring the lower uterine segment thickness (LUS) by ultrasound between the 35 and 38 week of pregnancy, to estimate the risk of uterine rupture [28, 29, 49]. Findings indicated: (1) the combination of abdominal and endovaginal ultrasounds allowed an optimal detection rate for uterine scar defect; [50, 51], (2) a LUS thickness < 2.0 mm represent a higher risk of uterine rupture (>1%), a LUS thickness ≥ 2.0 and < 2.5 mm represent a moderate risk of uterine rupture (around 0.5%), and a LUS thickness ≥ 2.5 mm represent a low risk of uterine rupture (<0.3%); [49] and (3) the use of LUS thickness in clinical practice was associated with no symptomatic uterine rupture among 984 women who underwent a TOL after one prior cesarean delivery [49], compared to 0.36% in the meta-analysis of Guise et al. [16]. These results were confirmed by a recent meta-analysis on the effect of the LUS method on uterine rupture (OR = 0.19; CI 95% = 0.09 to 0.40) [29], supporting the hypothesis that the LUS method, performed between the 35 and 38 week of pregnancy, is currently the most effective instrument to predict uterine rupture in women with previous low-transverse cesarean [28, 29, 49].

The availability of new, validated and standardized tools may facilitate the decision-making process as they may assist in the prediction of TOL success as well as of uterine rupture [24, 28, 29, 49]. However, their effectiveness in leading to an optimal selection of women for a TOL remains uncertain. This trial was designed to assess whether a multifaceted intervention based on the implementation of these tools into clinical practice (PRISMA program) will reduce the rate of major neonatal morbidities among women with one prior cesarean delivery.

### Hypothesis

Our primary hypothesis is that women exposed to the PRISMA program (Process for decision-making, RISk assessment and MAnagement in obstetrics) will have a rate of major perinatal morbidity, as measured in the hospitals during the intervention period, that is reduced by 25% in comparison with the control group (relative reduction). Secondary hypotheses are that the PRISMA program will: (1) reduce the rate of major maternal morbidity, (2) reduce the rate of minor maternal and perinatal morbidity, and (3) increase the rate of VBAC.

## Methods/Design

### Study design

**Fig. 1:**
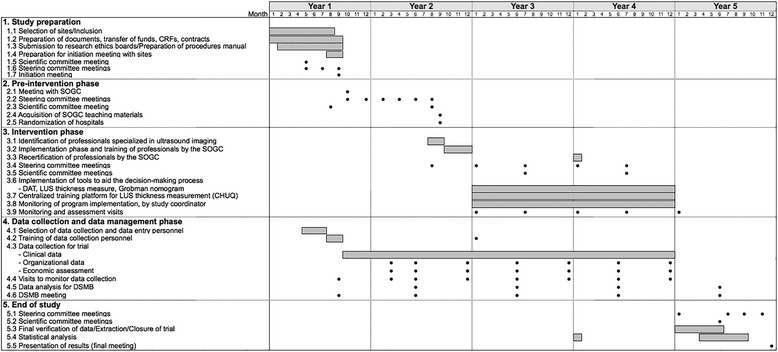
SPIRIT schedule for trial preparation, pre-intervention, intervention and assessments

This is a non-blinded, multicentre, stratified, cluster-randomized, parallel-group trial in which hospitals are the units of randomization and patients are the units of analysis. Randomization will be stratified according to level of care (community, regional, or tertiary hospital). The study included a 1-year pre-intervention (baseline) period, a 5-month implementation period, and a 2-year intervention period (Fig. 1).

### Inclusion and exclusion criteria

All hospitals entered the trial in April 2016. The study is conducted in 40 out of 48 eligible public hospitals in the province of Québec. To be eligible, each center was required to offer TOL after cesarean in their population; to have a minimum of 300 deliveries in the previous year; to have functional operating rooms; to have access to a high-performance sonographic device; and at the time of recruitment, to have no recent or ongoing quality-improvement programs specifically designed to target women with one prior cesarean delivery. Hospitals where LUS thickness measurement was already available in routine clinical practice and will remain available during the study were excluded from the trial.

At the patient level, women with only one prior cesarean delivering at one of the participating centers at a minimum of 24 weeks with one newborn who weighs at least 500 g at delivery are included in the analyses. Women with delivery or miscarriage before 24 weeks of gestation, with more than one previous cesarean delivery or with multiple gestations will be excluded from the trial.

### Intervention group activities

Intervention group activities will start in April 2017 and will end in August 2019. The PRISMA program, which will be implemented at the hospital level in the intervention group, targets physicians and nurses involved in the decision-making process for an ERC or a TOL among women with one prior cesarean delivery. The PRISMA program consists of: (1) a decision-aid tool to support women’s decision making in the choice of the mode of delivery (DAT), (2) standardized risk assessment tools leading to an optimal selection of women eligible for a TOL, i.e.: (a) an estimation of the chance of vaginal birth after cesarean (VBAC) success (Grobman nomogram), and (b) an estimation of the risk of uterine rupture using ultrasound measurement of lower uterine segment (LUS) thickness, and (3) a professional training program from the Society of Obstetricians and Gynaecologists of Canada (SOGC) to describe an optimal management of delivery and labor dystocia for a TOL. No financial incentive will be provided.

#### Professional training program from the SOGC

The 5-month implementation period will focus on implementing the PRISMA program in each of the 20 hospitals randomized to the intervention group. In each centre receiving the intervention, health professionals in obstetrics (physicians, nurses qualified in obstetrics, and midwives, if available) will be invited to participate in an onsite 1-day training workshop, provided by certified instructors from the Society of Obstetricians and Gynaecologists of Canada (SOGC), on best practices regarding intrapartum management among women with one prior cesarean delivery, as well as on the PRISMA clinical prediction tools. Using SOGC clinical practice guidelines and algorithms, health personnel will be trained on the following topics: prolonged pregnancy, management of the first and the second stages of labor, induction and stimulation of labor, fetal health surveillance, use of the WHO partograph [52], and assisted vaginal delivery. The SOGC training program will also include a workshop on the criteria to be assessed and discussed with the women during the decision-making process, as well as on the clinical use and interpretation of the Grobman prediction tool and of the ultrasound evaluation of the risk of uterine rupture. The training program will also sensitize participants to maternal factors contributing to the choice of the mode of delivery. Finally, in each hospital receiving the intervention, one or two technicians and physicians, with expertise in sonography, will be identified and invited to receive additional 1-day training in sonographic techniques related to the evaluation of the risk of uterine rupture. A half-day of recertification will be scheduled at the beginning of the second year of the intervention period. The entire training program will be overseen by the SOGC, which possesses extensive expertise in the field of professional training [53]. Details of the training program and algorithms will be available after the trial (September 2019) upon request by email to the corresponding author (NC).

#### Implementation of clinical tools leading to an optimal selection of women eligible for a TOL

After the 5-month implementation period, physicians from the intervention hospitals will be asked to use the three clinical tools of the program that assess the women’s initial preferences (DAT), the risk of uterine rupture (LUS) and the chances of TOL success (Grobman nomogram) for a period of 2 years from September 2017 to August 2019 (intervention period).

##### Decision analysis tool

The DAT used in this trial, entitled “Giving Birth after Cesarean Section: Making an Informed Choice”, has been adapted from the A. Shorten Birth Choices study [43], validated in Quebec in 2011 [54], and has received a 96% ranking on the International Patient Decision Aid Standard (IPDAS) assessment scale [55, 56]. The DAT is divided into two sections: (A) an Information section, providing a description and an explanation of the risks and benefits of each option, and (B) an Exercise section, allowing women to clarify and summarize their values and preferences with their physician about the choice of the mode of delivery, indicating what suggestions are important and how important these suggestions are (not important, important, very important). The DAT will be distributed between 12 ^0/7^ and 34 ^6/7^ weeks of pregnancy by the treating physician, preferentially early in the pregnancy, to all women with one prior cesarean delivery during their follow-up visit. The physician will ask the women to read the DAT carefully and complete the Exercise section, so that they will be able to discuss their preferences during their next appointment.

##### Grobman nomogram

Antepartum assessment of the chances of TOL success among women with one prior cesarean delivery will be conducted by the treating physician between 12 ^0/7^ and 34 ^6/7^ weeks of pregnancy, during one of the patient’s follow-up visits [25]. The treating physician will use the Grobman nomogram, derived from logistic regressions, which generates probability of VBAC success and a confidence interval of 95% [25–27, 46, 47]. This tool is based on maternal characteristics available at the time of the first prenatal visit, i.e. maternal age, Body Mass Index, prior vaginal delivery, prior successful VBAC, and recurrence of indication of prior cesarean delivery [25]. The assessment will then be completed after 36 ^6/7^ weeks of pregnancy, according to the maternal characteristics available at the time of admission, i.e.: estimated gestational age at delivery; hypertensive disease of pregnancy; labor induction; effacement; dilation; and station [26]. Results related to VBAC success and risk level will be discussed with each woman during their pregnancy and upon admission for delivery.

##### Ultrasound assessment of the risk of uterine rupture (LUS clinic)

The treating physician will routinely refer eligible women to the LUS clinic between 35 ^0/7^ and 38 ^6/7^ weeks of pregnancy. Each woman will meet with a nurse who will verify her eligibility, and will obtain her consent to the LUS. Eligibility criteria for the sonogram include: one prior cesarean delivery, and no uterine incision or laceration other than lower transverse; no multiple gestation; no known lethal congenital abnormalities; no preterm rupture of membranes; and no labor at the time of sonography. After ensuring that the woman has a full bladder, a trained ultrasound technician or a physician will measure the lower uterine segment (LUS) thickness by abdominal and endovaginal ultrasounds. The lower uterine segment will be swept in a sagittal cut from the right extremity to the left extremity, with the probe placed to detect the thinnest area of the lower segment. The technician/physician will also record the amniotic fluid index, the biparietal diameter, the cranial and abdominal circumference and the femoral length, in order to estimate the fetal weight. For each of the abdominal and endovaginal ultrasounds, the technician/physician will capture three pictures and a 15-s film (2D-ultrasound in real time).

Each 15-s film will be then immediately sent to a centralized platform in Québec in order to allow an external validation of each film in less than 1 h by experienced experts in measurement of the LUS thickness. This continuous training platform will accelerate the learning curve among health professionals in hospitals from the intervention group. The technicians of the centralized platform will capture three total measurements of the thinnest lower segment for each of the abdominal and endovaginal ultrasounds, based on the two 15-s films, and then send the results to the physician of the LUS clinic at the referring hospital. Once the measurements have been taken and the results of the external validation in Québec are available, the physician responsible of the LUS clinic, who has also been trained, will validate the exam and reproduce it immediately, as required. Only the thinnest measurement, from among the six measurements taken via the abdominal and endovaginal ultrasounds, will be used. For each professional, a continuous quality assessment of the measurement, based on objective criteria, will be conducted during the first 30 measurements of the LUS thickness. Additional training could be considered depending on the performance of each professional [28, 50, 51, 57–59].

The physician in charge of the LUS clinic will complete a consultation form to be sent to the treating physician. The treating physician will be informed of the estimated fetal weight (EFW) and the risk of uterine rupture and will discuss the results with the woman. According to lower uterine segment thickness, women will be classified into three risk categories for uterine rupture: high risk (< 2.0 mm), intermediate risk (≥ 2.0 mm and < 2.5 mm), and low risk (≥ 2.5 mm). If the EFW falls below the 95th percentile and the LUS thickness is ≥ 2.5 mm, the treating physician will be informed that the risk of uterine rupture is < 0.3% in the absence of labor induction with unfavorable cervix, and that a TOL should be recommended in the absence of other contraindications [16, 28, 29, 45, 60–62]. If the LUS thickness is < 2.0 mm, the treating physician will be informed that the risk of uterine rupture is > 1%, and that a TOL should not be considered except in exceptional circumstances (e.g. admission at full cervical dilatation). If the LUS thickness is ≥ 2.0 and < 2.5 mm, the treating physician will be informed that the risk of uterine rupture is intermediate (around 0.5%), and that a TOL could be considered if no other particular conditions are observed. The exact LUS thickness, however, will not be conveyed to the patient or treating physician.

### Control group

No intervention from the PRISMA team is planned for the control group. In order to assess contamination bias, quality-improvement programs will be reviewed annually in control hospitals.

### Study outcomes

The primary outcome is the major perinatal morbidity rate. The composite risk of major perinatal morbidity includes: (1) In utero, intrapartum and neonatal death, defined as the fetal death in utero, during labor or prior to 28 days of age (excluding lethal congenital abnormalities); (2) APGAR score at 5 mn < 4; (3) Metabolic acidosis (umbilical arterial pH < 7 + base excess ≤ −12 mmol/l); (4) Major trauma (skull fracture, subdural/subarachnoid haemorrhage, brachial plexus injury, spinal cord injury, major genital injury, paresis/paralysis at discharge); (5) Intracerebral/intraventricular haemorrhage (grades 3 and 4); (6) Periventricular leukomalacia; (7) Seizure (occurring from delivery to discharge); (8) Invasive mechanical ventilation with endotracheal intubation; (9) Major respiratory morbidity (bronchopulmonary dysplasia treated with oxygen or ventilation at 36 weeks post menstrual age or at 28 days of life, persistent pulmonary hypertension of the newborn, pneumothorax, pulmonary haemorrhage, hyaline membrane disease requiring mechanical ventilation); (10) Necrotising enterocolitis, stage 2 and 3; 11) Hypoxic-ischemic encephalopathy (APGAR 5mn < 4 + pH < 7 + base excess < −12 mmol/L + seizure); (12) Proven neonatal sepsis/infection (positive blood or cerebrospinal fluid culture); and (13) Hypotension requiring vasopressor support. The binary primary outcome (0; 1) event is triggered by occurrence any of the above morbidities. Value 1 is attributed if at least one of the above conditions is met.

Secondary outcomes include:

1. *The rate of major maternal morbidity:* this indicator is measured with a composite factor of maternal morbidity including: (1) maternal death (≤ 6 weeks postpartum); (2) hysterectomy; (3) proven thromboembolic complications (thrombophlebitis, pulmonary emboli); (4) admission to intensive care unit for ≥ 4 days; (5) acute pulmonary edema/cardiogenic shock; (6) sepsis with proven bacteremia (septicemia); (7) injury/lacerations to internal organs, requiring surgical repair (bladder, bowel, large vessels); (8) symptomatic uterine rupture (complete rupture of the uterine wall with extrusion of amniotic fluid, placenta, or fetal limb outside the uterine cavity, or with rupture of the bladder, requiring surgical repair)
2. *The rate of minor maternal morbidity:* this indicator is measured with a composite factor of maternal morbidity including: (1) blood transfusion; (2) postpartum hemorrhage requiring surgical procedure; (3) perineal tear of third or fourth degree; (4) cervical lacerations requiring repair; (5) dehiscence of skin wound (C-section or perineal); (6) puerperal fever (≥ 38 °C postpartum); (7) postpartum infection requiring drugs or surgery (infection of the incision (C-section or perineal), uterus (e.g. endometritis), urinary tract (≥ 10^5^ CFU/ml), respiratory tract (e.g. pneumonia)); (8) major gastrointestinal complications (intestinal occlusion); (9) obstetric anesthesia complications (neurological, cardiovascular, respiratory); (10) length of postpartum hospital stay ≥ 7 days; (11) admission to intensive care unit for less than 4 days; and (12) readmission to hospital within 40 days of childbirth
3. *The rate of minor perinatal morbidity:* this indicator is measured with a composite factor of perinatal morbidity including: (1) minor cardiorespiratory complications (cardiac rhythm disorders, delayed amniotic fluid clearance, transient tachypnea of the newborn); (2) non-invasive mechanical ventilation; (3) admission to the intensive care unit for less than 4 days; (4) oxygen therapy; (5) suspicion of neonatal infection (culture-negative); (6) APGAR score ≥ 4 and < 7 at 5 min; (7) acidosis (umbilical artery pH ≥ 7 and < 7.20); (8) minor trauma (lacerations, limb fracture, clavicle fracture); (9) transfusion in first 24 h; and (10) weight loss > 10% in first ten postnatal days
4. *Rate of trial of labour (TOL):* this is the number of women attempting a vaginal delivery divided by the total number of women with one previous C-section
5. *Rate of VBAC:* this is the number of vaginal deliveries divided by the total number of women with one previous cesarean delivery

### Randomization and allocation

In April 2017, after the 1-year baseline period, hospitals will be randomly assigned either to the intervention group or the control group. Randomization will be stratified according to level of care of each hospital (community, regional, or tertiary hospital). To avoid imbalance in the size of the two groups, we will use computer-generated, blocked randomization within each stratum, with blocks consisting of four centers or, for strata with fewer than eight hospitals, two centers. The names of participating hospitals will be coded and the randomization will be generated by an independent statistician blinded to group allocation. The Data Safety Monitoring Board will receive the results of the randomization and the key code to ensure validity of the process and reveal the distribution of both intervention and control groups to the coordination team. Local investigators at each hospital will then immediately be informed of the assignment status of their hospital.

### Data collection and management

#### Data collection

Clinical data are collected independently of the intervention. Subjects will be initially screened by the medical archives department staff in every participating hospital, and then will be validated by the data collectors according to the study inclusion and exclusion criteria. Computerized data collection will be performed on-site using Dacima Clinical Suite from Dacima Software Inc. [63] The electronic case report form includes individual clinical information about the mother and the newborn, namely maternal characteristics, prenatal care, labor and delivery and maternal and neonatal complications. In-hospital data will be abstracted by trained research nurses or medical archivists from the medical records of mothers and newborns at least 3 months after delivery; data will be abstracted in the same way at both intervention and control hospitals from April 2016 to March 2017 (baseline period) and from September 2017 to August 2019 (intervention period). Data collectors will be aware of the randomization assignments but will not be involved in outcomes assessment.

#### Data management

The Dacima Clinical Suite and the trial database are managed by the Data Management Team located at the Research Center of the CHU de Québec (Laval University, Québec City, QC) and hosted on secure servers in *Dacima software* facilities (Montreal, QC). Until the end of the trial, access to the clinical database will be restricted to the Data Management Team. All steps involved in the management of clinical data will be annually monitored by an independent Data Safety Monitoring Board (DSMB).

#### Quality control of data

Four levels of control have been set up. The first level represents the data entry controls included in the Dacima software (bounds, branching logics, associations between variables). The second level is a random case review of around 5% of all medical records by a second data collector. In addition, all cases of major perinatal and maternal morbidity will be systematically monitored for quality. The third quality control level targets specific cases of perinatal and maternal morbidity for which a consensus between the collectors and the data management team could not be reached. These cases will then be submitted to a committee of experts (obstetricians and neonatologists) for further study. Finally, the Data Management Team will assess the data completeness and quality directly in the database (fourth level of control). Discrepancies will be resolved through onsite visits and queries sent to data collectors.

### Statistical analysis plan

#### Sample size and power calculation

The sample size was calculated to maximize statistical power while minimizing the number of clusters [64]. Sample size calculations were based on a cluster RCT design [65], stratified by level of care (community, regional or tertiary care hospitals), to detect a relative reduction of 25% in major perinatal morbidity in hospitals of the intervention group compared with those of the control group. To account for clustering by hospital, we assumed an intraclass correlation coefficient of 0.001, estimated from the QUARISMA Trial data, representing 70% of deliveries in Quebec in 2010–2011 [64]. We calculated that we would have to enroll 40 hospitals in the study, with an expected total of 7360 women with one prior cesarean delivery per year (22,080 women for the 1-year baseline period and the 2-year intervention period), to achieve a power of 80% to detect a 25% relative reduction with the intervention in the major perinatal morbidity rate, assuming a baseline rate of 4.5% for the three strata, at a two-sided alpha significance level of 0.05 [64, 66].

#### Type of analysis and handling loss-to-follow-up

Analyses will be conducted in intention-to-treat, i.e., each patient will be analyzed in the hospital to which she will be admitted and each hospital will be analyzed in the group to which it will be assigned by the randomization. If a hospital decides to withdraw from the trial after the randomization, data collection will continue until the end of the trial, according to the commitment made by the hospital authorities at the time of inclusion. This hospital will not be excluded from the analyses.

#### Statistical analysis

For reducing contamination bias, hospitals are the units of randomization and patients are the units of analysis [64, 67]. In the primary intention-to-treat analyses, we will assess the effect of the intervention on the rate of major perinatal morbidity using the multivariable generalized-estimating-equations extension of logistic regression, with an exchangeable covariance matrix, to account for the clustering of women within hospitals [68]. Changes in the risk of major perinatal morbidity in the two study groups between the 1-year baseline (pre-intervention) period and the 2-year intervention period will be compared with the use of an adjusted odds ratio (with 95% confidence intervals) for the interaction between group (intervention vs. control) and time period (intervention vs. baseline) [64, 65]. The adjusted odds ratio for interaction will be estimated with the use of data on women who were delivered during the baseline period or the intervention period and measures the intervention effect with the difference-in-differences approach [69, 70], which is adapted for generalized-estimating equation analyses of clustered binary outcomes [65]. In each group, the change over time, from the baseline to the intervention period, will be measured by the respective odds ratio, i.e. the exponent of the difference between log odds of cesarean delivery in intervention versus baseline periods. Then, additional change over time in the intervention group, relative to the concurrent change in the control group, will be estimated by the difference between group-specific differences. The exponent of the resulting difference-in-differences in log odds provides the adjusted odds ratio, with 95% CIs, for the interaction between group (intervention vs. control) and time (intervention vs. baseline), which will be used as the main measure of the intervention effect [64, 69, 70]. Two-tailed *P* values of less than 0.05 will be considered to indicate statistical significance.

All primary analyses will include adjustments for prespecified potential maternal, perinatal and institutional risk factors associated with major perinatal morbidity [64]. To conform to the intention-to-treat approach, all eligible women who deliver at participating hospitals will be included in the analyses. For adjustment variables for which less than 1% of the data are missing, we will use random imputation (performed on the basis of the observed distributions of the imputed variable and of highly correlated covariates). Variables for which more than 1% of the data are missing will be excluded from the analyses. Randomization will be stratified by level of care in hospitals. To assess whether the intervention effect will vary according to the level of care, we will test the corresponding three-way interactions: level of care × intervention × time. Subgroup-specific intervention effects will be reported for outcomes with significant three-way interactions (two-tailed tests, with *P* value of less than 0.05 will be considered statistically significant) [69, 70].

Secondary outcomes will be analyzed by means of methods similar to those used for the primary outcome. Subgroup analyses for preterm and term birth will be also planned to take into account major neonatal morbidity related to preterm birth and not related to the mode of delivery. If the generalized-estimating- equations models do not converge, the intervention effect will be estimated with the use of multivariable logistic model, which do not account for within-hospital clustering; to correct for the resulting underestimation of the standard errors, a conservative *P* value of less than 0.001 will be used [68, 70]. All analyses will be performed with the use of SAS software, version 9.4 by an independent team whose members will be unaware of the group assignments.

#### Economic evaluation

Cost-effectiveness analyses will take the perspective of the publicly funded health care system [71]. The time horizon will capture hospital-based costs and clinical events for mothers and neonates from labour onset to a minimum of 3 months postpartum. Resource use will be identified and measured from patient charts and valued using standardised government sources, following recommended methods for costing in the Canadian context [72]. A discount rate of 5% will be applied, and results will be compared to two scenarios implementing alternative discount rates of 3% to facilitate comparisons with other jurisdictions and 0% to explore the effect of discounting [71].

The main analysis will be an ITT analysis including all trial participants. To assess intervention impact, we will model changes in a composite index of major perinatal morbidities and costs in the two study groups between the 1-year baseline period and the 2-year intervention period using adjusted regression coefficients (with 95% confidence intervals) for the interaction between group (intervention or control) and time period (intervention or baseline) [64, 70]. Costs and effects will be modeled jointly using bivariate multilevel linear models that explicitly recognize potential correlations between the bivariate outcomes (major perinatal morbidities and costs) at individual and cluster (hospital) levels [73]. Crude and adjusted statistical models will be also estimated. Crude models will include study group (intervention vs. control), time period (baseline vs. intervention) and the interaction between study groups and time period. Adjusted models will use precisely the same variables for adjustment of confounding, as the main trial. All variable definitions will be identical to those used for the main trial. To address potential heterogeneity in cost-effectiveness, analyses will be repeated for pre-specified subgroups of interest (level of care, prematurity).

For each analysis, the incremental cost-effectiveness ratio (ICER) statistic will be calculated as the ratio of additional costs per reduction in major perinatal morbidities, and estimate its confidence interval using the Bayesian Markov Chain Monte Carlo (MCMC) method [74]. As interpretation of the ICER can be challenging, the Incremental Net monetary Benefit (INB) will be also calculated using a range of reasonable values to represent societal willingness to pay (*ʎ*) [75, 76]. Analyses will be also conducted to explore the implications of stochastic, parameter and structural uncertainty, as well as heterogeneity [77]. We will use the Bayesian MCMC method to ascertain the joint uncertainty of the estimands, incremental costs and effects [78], and cost-effectiveness planes plotting the values of incremental costs and effects stored in the Markov chains to present the joint uncertainty of the estimands [74]. To explore potential heterogeneity we will repeat all analyses over subgroups based on hospital level of care [74]. Results of the uncertainty analyses will be graphically presented in a cost-effectiveness acceptability curve (CEAC) that depicts the uncertainty about the new intervention being cost-effective as a function of societal willingness to pay (*ʎ*) [77–80]. Finally, we will decompose costs into categories reflecting the secondary trial outcomes to identify drivers of change. Cost-effectiveness models will be implemented in MLwiN [81] within a Stata environment [82]. An independent team not involved in the conduct or analysis of the main trial will perform the economic analyses.

### Potential limitations of the trial

Our study could have some limitations. Since hospitals will be the unit of randomization, and the number of deliveries varies across hospitals, there could be differences in the distribution of hospital characteristics across groups at baseline. These differences will be adjusted a priori in multivariable analyses. Because of potential intra-cluster variation among hospitals, the trial results could be driven by a few hospitals. Consequently, adjusted, center-specific multivariable analyses will be also conducted to assess changes over time in the major perinatal morbidity rates in each individual hospital. Furthermore, the intervention may not be fully implemented in each intervention hospitals. Finally, because we will assess a complex, multifaceted intervention, it will not be possible to determine which of its components will be primarily responsible for any observed effect.

### Ethical issues

#### Ethics committee approval

The trial has been approved by the Ethics Committee of the CHU de Québec-Université Laval in Québec, Canada and of all 40 participating hospitals (MP-20-2016-2718; MP-20-2017-3220). The PRISMA trial is registered on the ISRCTN registry under the number ISRCTN15346559 (http://www.isrctn.com/). The results of the trial will be reported according to the CONSORT Statement (SPIRIT Checklist, Additional file 1).

#### Information and consent forms

The participating hospitals are included on the basis of an informed consent form signed by the head of obstetrics. The informed consent form specifies that: (1) the site can withdraw from the program at any time; (2) the data collection will continue until the end of the trial, even if a site withdraw from the project; and 93) that the PRISMA program will be offered to control group hospitals at the end of the trial if the intervention is proven effective. Collection of clinical data from medical records is authorized by the director of professional services in each participating hospital and does not require patient consent. During the intervention period, the involvement of patients will be sought with the use of the decision analysis tool (DAT) and for the LUS thickness measurement. The DAT includes an implied consent which states that filling it and returning it to their physician represent a women’s consent to use the DAT. The authorisation for an additional ultrasound measurement (LUS thickness) will be obtained by a written information and consent form.

#### Interim analyses and stopping rules

An independent Data Safety and Monitoring Board (DSMB) has been established, made up of three international experts in epidemiology and biostatistics, reproductive health research, and obstetrics and gynaecology. It will be responsible for ensuring the safety of the trial and for monitoring the progress of the research according to the protocol. Serious adverse events (maternal and perinatal death, perinatal asphyxia, hemostatic, hysterectomy, uterine rupture) will be monitored annually during the intervention by the DSMB, blinded, for all patients in the study. To assess the effectiveness of the program at the end of the first year of the intervention period, an intermediate analysis will be planned using Peto criteria (*α* = 0.001), which ensure that a total type-I risk of error, for the final analysis, of 0.05. The DSMB may decide to stop the trial for the following reasons: detection of adverse events, poor data quality, low levels of implementation, high contamination in the control group, fraud, and new information that would rule the trial unnecessary, futile, or unethical.

### Sponsor and project administration

The project was approved by the Canadian Institutes of Health Research (CIHR) and funded in July 2015. The trial begun in April 2016. The funds are managed by the Université Laval. The multidisciplinary PRISMA research group is composed of researchers and physicians from Canada, United States and France with an international expertise in maternal and perinatal health, VBAC, clinical research, RCTs, biostatistics, improvement of quality of care and assessment of health care programs. The PRISMA intervention program is coordinated by a group of experts in maternal health and VBAC, in collaboration with the Society of Obstetricians and Gynaecologists of Canada (SOGC), the Ministère de la santé et des Services Sociaux du Québec (MSSSQ), the Association des Obstétriciens Gynécologues du Québec (AOGQ), the Association des Omnipraticiens en Périnatalité du Québec (AOPQ), and the Canadian Association of Perinatal and Women’s Health Nurses (CAPWHN). The management of the program implementation in hospitals of the intervention group and the conduct of mid-intervention audits will be carried out by the administrative coordinator of the trial. The data management and quality control are assumed by the scientific coordinator, who is also responsible for sending data to the DSMB for annual assessment of the safety of the trial and compliance with the protocol and timeline. The data collection and the intervention are two independent processes. A cross-disciplinary Steering Committee, with extensive international expertise in RCT, VBAC, quality of care improvement and health care program assessment, meets at least every 3 months to coordinate the trial, taking into account DSMB recommendations.

## Discussion

The PRISMA trial is a phase III cluster randomized controlled trial to assess the effectiveness of a multifaceted intervention aiming to reduce severe perinatal morbidity in women with one previous cesarean delivery. The PRISMA Program is designed to facilitate: 1) women’s decision-making process, using a decision analysis tool (DAT); 2) an estimate of uterine rupture risk during TOL using ultrasound evaluation of low-uterine segment thickness; 3) an estimate of chance of TOL success, using a validated prediction tool; and 4) the implementation of best practices for intrapartum management.

The DAT will be available in each intervention hospital in the clinic waiting rooms and distributed by the treating physician early during the pregnancy to help women make an enlightened choice. This tool will be also discussed with the physician during the next appointment. An onsite professional training program from the SOGC including decisional algorithms about selection of women for a TOL, chance of VBAC success, optimal management of delivery and labor dystocia among women with one prior cesarean delivery, is also planned in each intervention hospital. This professional training will be planned several times in each hospital in order to be able to train a high number of health professionals (obstetricians and gynaecologist, family physician, nurses, midwives). Finally, several onsite ultrasound training are planned in each intervention hospital, during the 5-month implementation period, in order to train ultrasound health professionals to assess the risk of uterine rupture at 35 weeks (LUS technic) among women with one prior cesarean delivery.

An important challenge relies in the implementation of the program, which may differ in different hospitals. In this Trial, a high level of implementation will be ensured by the support of local partners and healthcare professionals identified in intervention hospitals, by continuous internal assessments for compliance, by an annual site monitoring visit or more if required, and by a centralized platform in Québec allowing an external validation of each LUS thickness measures. This real time validation will ensure a constant follow up with participating physicians.

The PRIMSA Program aims to provide the right intervention for the right woman at the right time, and will contribute to reduce the perinatal morbidity among women with a prior cesarean delivery. This trial will provide a high level of evidence regarding the choice of the optimal mode of delivery, as well as the management of delivery in women with a prior cesarean.

### Trial status

At the time of submission, participating hospital have been enrolled, randomized and patient recruitment begun in one hospital.

## Additional file

Additional file 1: SPIRIT 2013 Checklist: Recommended items to address in a clinical trial protocol and related documents. (PDF 100 kb)
